# *Nummi* Digitali: A pioneering multimodal platform for numismatic heritage

**DOI:** 10.1371/journal.pone.0332151

**Published:** 2025-10-03

**Authors:** Lavinia Sole, Dario Giuffrida, Elisa Chiara Portale, Francesco Armetta, Massimiliano Perri, Oreste Adinolfi, Maria Luisa Saladino, Rosina Celeste Ponterio

**Affiliations:** 1 Dipartimento Culture e Società, Università degli Studi di Palermo, Viale delle Scienze, Palermo, Italy; 2 Consiglio Nazionale delle Ricerche, Istituto per i Processi Chimico Fisici, Messina, Italy; 3 Dipartimento di Scienze e Tecnologie Biologiche Chimiche e Farmaceutiche-STEBICEF, Università degli Studi di Palermo, Viale delle Scienze, Palermo, Italy; 4 Webgenesys S.p.A., Via Rodi, Roma, Italy; 5 Zeiss Group Via Varesina 162, Milano, Italy; Flinders University, AUSTRALIA

## Abstract

This work presents a pioneering multimodal platform designed for the study, cataloging, and dissemination of numismatic heritage: “*Nummi* Digitali”. This ecosystem enables a web-based structured recording of coin data according to Italian ministerial standards, international interoperability protocols, and Linked Open Data (LOD) principles. The back end facilitates rigorous cataloging and integration of high-resolution 2D imaging, 3D metric models, and archaeometric data—including non-invasive XRF analysis of alloy composition—while the front-end provides interactive access for researchers and the public. The platform’s architecture supports multilevel user interaction, offering advanced analytical tools for specialists and accessible visualization for broader audiences. For the first time, a unified digital infrastructure connects traditional numismatic metadata with metrological, physical and chemical data, establishing a new paradigm in digital numismatics. Initially, the system was tested on a core collection of Greek, Punic, and Roman coins belonging to the “A. Salinas” Regional Archaeological Museum in Palermo (Italy), achieving significant outcomes in terms of scientific analysis, historical contextualization, and public engagement. Its scalable and standards-compliant design positions “*Nummi* Digitali” as a transformative model for interdisciplinary research and digital cultural heritage.

## Introduction

Ancient numismatics has long been a cornerstone for understanding economic and social systems, trade networks, political structures and cultural exchanges across ancient civilizations. Numismatic research has always been based on the cataloguing of coins, which is essential for understanding the artefact: it involves the visual analysis of the specimens, the comparison with archive documents (inventory registers; excavation documentation; documents on provenance) and the use of technical data collection tools and two-dimensional photographic reproduction.

Over the last twenty years, digital technologies have innovated numismatic research. In this framework, databases have proven to be indispensable tools to facilitate cataloging, analysis and comparison between series belonging to multiple collections or archaeological contexts, collaborative research, and to improve public accessibility to numismatic data. In line with this paradigm shift, cultural and academic institutions are leading several digitization initiatives with the aim of creating online databases to make numismatic collections and complexes accessible. Among recent initiatives, both thematic and excavation-based projects have created online repositories to catalogue coin finds, from regional hubs and site-specific archives to comprehensive issues databases spanning Greek, Roman, Byzantine, and medieval contexts (references for each database can be found in S1 Appendix). These platforms often stem from university-led projects or museum consortiums and provide records, high-resolution imagery, and, in some cases, basic contextual metadata. At the same time, specialized portals document hoards, site-specific assemblages, and individual institutional collections, linking disparate holdings into a broader network of open data.

While such repositories have substantially improved accessibility and collaborative research, they operate under a variety of cataloging standards and technical frameworks—resulting in variable data quality, inconsistent metadata schemas, and limited cross-platform interoperability. For instance, European and American databases utilize the Nomisma.org [1–3] framework, which is instrumental in standardizing numismatic metadata and creating a structured semantic web for coin data, whereas Italian numismatic databases, aligned to the Ministry of Culture’s guidelines, adhere to national cataloging frameworks, structured by the Istituto Centrale per il Catalogo e la Documentazione (ICCD) [4–6]. Thus, both are based on distinct, non-aligned cataloging standards and are almost always lacking in:

- Archaeological find data, essential to know the relevant contexts and to enhance the understanding on the function and circulation duration of the coin.
- Georeferenced data, which provides the opportunity to analyze numismatic evidence in relation to its place of discovery or production.
- 3D representations, which can capture the morphological details, fundamental for the study of iconography, production technique and voluntary alterations of coins.
- Archaeometric data, which allow analyses such as metal composition, trace elements derived from spectroscopic and isotopic methods).
- Dynamic and interactive visualizations of the coins for both specialized researchers and the wide public.

These gaps have highlighted the need for an integrated and interdisciplinary platform functioning as a digital ecosystem. “*Nummi* Digitali” sets a new standard for numismatic research, but also for museum management and public involvement, by introducing a powerful framework that integrates a structured database for cataloging data, information relating to the context of the coin discovery, high-resolution images, 3D metric models and results of archaeometry investigations. The system, open access and web-based, is specifically designed by specialists for specialists, but is also suitable for broader use.

## Materials and methods

To assess the functionality of the *Nummi* Digitali platform, a pilot study was conducted, initially using a group of Greek, Punic, and Roman coins from the “A. Salinas” Regional Archaeological Museum in Palermo (Italy), chosen from the collection’s most representative items, considering both their chronological span (from the Archaic to the Late Antique period), their place of production (mints predominantly located in Sicily) and unique technical characteristics (cast, struck, suberated, of dubious authenticity) (Table 1).

**Table 1:** List of first coins underwent a comprehensive digitization process, encompassing cataloging, digital twins creation, and chemical analysis.

Inv. No.	Mint/ authority	Obverse	Reverse	Chronology	Metal; weight; denomination; diameter; axis	Provenance
26450	Selinus	Leaf of *selinon*	Incuse square divided into twelve triangular segments	540/520–510/500 BC	AR; 8,50; suberated didrachm; 22;/	Hoard IGCH 2067
26169	Selinus	Leaf of *selinon*	Leaf of *selinon* in incuse square	510−500 BC	AR; 8,84; didrachm; 22; 12	/
26259	Leontinoi	Naked rider on horse running r.	ΛΕΟΝΤΙΝΟΝ. Lion’s head right; around, four barley grains.	Around 470 BC	AR; didrachm; 8,52; 20; 8	San Martino Collection
26117	Gela	Charioteer driving quadriga r.; on background, Ionic column	CΕΛΑΣ. Forepart of a man-headed bull r.	465−450 BC	AR; 17.15; tetradrachm; 28; 3	Valenza Donation
64093	Akragas	Eagle standing l.	Crab; on base, four dots.	440−430 BC	AE; cast 15,66; trias; H 20; L 19; W 13	La Duca collection
26173	Selinus	ΣΕΛΙΝΟ-Ν-ΤΙ-[ΟΝ]. Apollo, holding bow, and Artemis in walking quadriga r.	[ΣΕΛ]Ι-ΝΟ-Σ. River-God *Selinos* standing l., sacrificing over altar; rooster on altar l., bull on basis and *selinon* leaf r.	440−420 BC.	AR; tetradrachm; 17,34; 27; 1	Valenza Donation
26249	Messana	Charioteer driving mule biga l.; *Nike* flying to crown charioteer; in exergue, two dolphins	ΜΕΣΣΑΝΙΟΝ. Hare running l.; below, head of Pan	412−408 BC	AR; 17.22; tetradrachm;25; 8	Valenza Donation
9509	Akragas	Eagle flying r. on fish	Crab; around, dots. Countermark: Head of Herakles	Late 5th- early 4th century BC.	AE; hemilitron; 15,70; 31; 9	/
26103	Panormos	Galloping quadriga r. with Nike in flight crowning the charioteer; in exergue, hippocampus and ṣyṣ.	Female head; around, dolphins.	410−390 BC	AR; tetradrachm; 16,97; 25; 11	Hoard IGCH 2119
9286	Carthago in Sicily	Forepart of running horse l. crowned by a flying Nike; on the l., grain of wheat	QRT-ḤDŠT. Palm	410−390 BC	AR; tetradrachm; 17,11; 25; 3	Purchase 1874
26078	Syrakousai	Galloping quadriga l. with Nike in flight crowning the charioteer; in exergue, panoply and ΑΘΛA.	Head of Arethusa l.; to l., Δ; around, dolphins.	406−397 BC	AR; decadrachm; 43,22; 34; 9	Hoard IGCH 2119
14537	Carthago	Female head l., crowned with ears of corn.	Horse standing r.	ante 300 BC	EL; shekel; 7,44; 19; 12	Purchase 1899
26232	Syrakosai, Hieron II	Diademed and veiled head of Philistis l.; on the r. thyrsus;	Nike drawing quadriga r; above, crescent moon; above, [Β]ΑΣΙΛΙΣΣΑΣ; on the r., Α; in exergue, ΦΙΛΙΣΤΙΔΟΣ.	218/217–214 BC	AR; tetradrachm; 13,57; 26; 1	Valenza Donation
10010	Panormos	Head of Ares r.	ΠΑΝΟΡ-ΜΙ-ΤΑΝ. Kore standing l. with patera and cornucopia.	90-50/40 BC	AE; 12,40; 26; 2; of dubious authenticity	Donation from the Prince of Scalea
65413	Rome, Hadrian	HADRIANV[S]-AVG COS III PP. Laureate head of Hadrian r.	FELICITATI-AVGVSTI. Ship l.	130 AD	AR; denarius; 3,34; 18; 9	Valenza collection
51735	Ravenna, Honorius	DN HONORS-VS PF AVG. Diademed, draped and armored bust of Honorius r.	VICTORI-A AVGGG. Honorius, draped, armored, standing r. with banner and a Victory on a globe to the l.; with the foot. crushes a prisoner; on the sides, mint mark, R-V; in exergue, CONOB	402-406 AD	AV; solidus; 4,43; 20; 6	/

^a^*References and archaeometric data for each coin can be found in Table A in* S2 Appendix*. Selection of coins from the “A. **Salinas” Regional Archaeological Museum of Palermo**. From the methodological point of view, the flowchart (*Fig 1) *provides a visual overview of the comprehensive digitization process that transforms a physical coin into a fully interactive and informative virtual coin, through the acquisition of data from metrology, imaging, archaeometry, and numismatics.*

**Fig 1:**
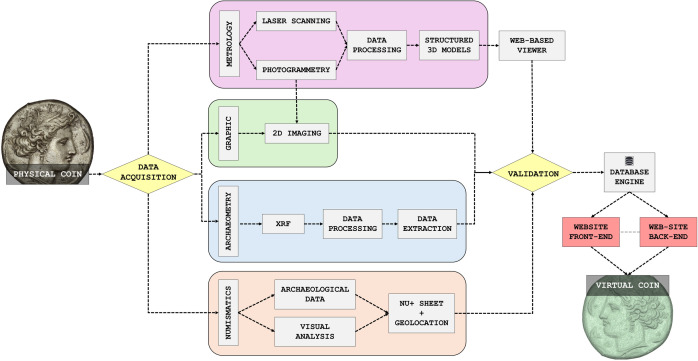
Workflow from physical coin to virtual coin in *Nummi* Digitali.

This museum, the oldest and most significant in Sicily, preserves a vast and historically significant numismatic collection, ranging from the earliest coin emissions of the mid-6th century BCE to modern-era specimens [7,8]. The collection includes a substantial number of Punic coins, minted by Carthage in Sicily, as well as exceptional specimens by the ”signing masters”, including some Syracusan decadrachms by *Kimon* and *Euainetos*. Each selected coins was cataloged according to the ”Scheda NU + ” standard to validate the platform’s core functionalities and underwent a comprehensive digitization process, encompassing cataloging, digital twin creation, and chemical analysis. All necessary permits were obtained for the described study, which complied with all relevant regulations.

The starting point is a tangible coin, which undergoes a multi-phase process of data acquisition and digital reconstruction. Data acquisition is divided into three distinct branches, each focusing on capturing different aspects of the coin’s characteristics. The first branch involves 3D data capture, employing both laser scanning and photogrammetry to create a detailed three-dimensional representation of the coin. These techniques allow the creation of highly accurate, textured 3D models that capture the nuances of the coin’s surface. Simultaneously, the second branch involves the cataloging of the coin’s historical and contextual information, adhering to standardized documentation practices. This is followed by a structured data validation to ensure the accuracy and reliability of the recorded information. This consisted of a multi-phase assessment involving both technical and curatorial evaluations: 1. cross-verification to ensure terminological and ontological consistency; 2. testing on a diverse coin sample from the museum to verify completeness, usability, and semantic adequacy of the metadata fields; iterative feedback involving domain specialists (archaeologists, numismatists, and data curators); 3. final approval for platform integration, ensuring compliance with FAIR principles and with national and international cataloging systems. In parallel, the third branch focuses on material analysis through XRF spectra collection, a non-invasive method used to determine the coin’s elemental composition. The chemical data obtained from the XRF analysis is then extracted and processed to make it suitable for integration with the 3D data and cataloging information. Specifically, the processed XRF datasets were linked to each coin’s metadata record through a dedicated field in the “Scheda NU+” schema and embedded into interactive 3D models in two formats: simplified textual annotations for genetal users and full spectral charts for experts.

Following data acquisition, the next phase emphasizes data integration and the development of a web platform to manage and present the digital information. The integration process involves combining the 3D models and material analysis data, ensuring that all elements are accurately aligned and validated. This stage is critical, as it requires balancing the complexities of data precision with the need for accessibility. The development of the web platform is divided into two main areas (see more in S3 Appendix):

- the back-end interface, tailored for experts to manage, edit, and validate the data;
- the front-end interface, designed for user interaction and visualization.

Once the data is fully integrated and the platform is established, the focus shifts towards the visualization of the virtual coin. This stage involves implementing interactive features that enhance the digital representation, allowing users to engage dynamically with the coin’s details, including zooming, rotating, and exploring specific annotations. The final product is then published on a website, making it accessible to both the general public and researchers, fostering a dual-level engagement that caters to both educational and scholarly needs.

The result is a seamless transformation from a physical coin into a virtual coin, representing a realistic digital replica that is both informative and accessible. This process not only preserves the physical attributes of the coin but also extends its reach, enabling a broader audience to interact with and study the artifact in a way that traditional display methods cannot match.

### Platform architecture

For the specific development of the back-end interface, we used the structure and ontology of the Scheda NU, a descriptive schema for numismatic cataloging established by the ICCD in 2004 [9]. This schema, comparable with the Numismatic Description Scheme (NuDS) [10] adopted by nomisma.org, is composed of multiple hierarchically structured sections and fields [11]. However, since the project required more flexibility to be adaptable to the web, to heterogeneous data, to modern directions of numismatic research and to a varied users’ base, several modifications were implemented:

- removing non-essential fields and addition of new research-specific fields (especially related to the contextual data), to adapt the database to a varied numismatic heritage (coins from historical collections or found in excavations), to the contextual approach of numismatic research and to web display;
- creation of specific ontologies, adapting traditional cataloging frameworks to semantic web paradigms [12];
- updates to field labels, introducing more intuitive equivalent terms from the 2017 ICCD Cross-cutting Regulation 4.00 [13].

The final customized schema, Scheda NU + , was validated by ICCD experts and published [14]. It is divided into 19 sections, covering all aspects necessary for numismatic research, including a specific section “Restorations and analysis” for detailed insights into the analysis of the metallic composition of coins. Each section is identified by a two-letter code, with fields arranged in cascading order. Dropdown menus and pop-up suggestions facilitate data entry using ICCD-formalized vocabularies and thus ensuring consistency and efficiency (see more in S3 Appendix). The back-end includes search functionalities by filters relating to numismatic keywords.

**Fig 2:**
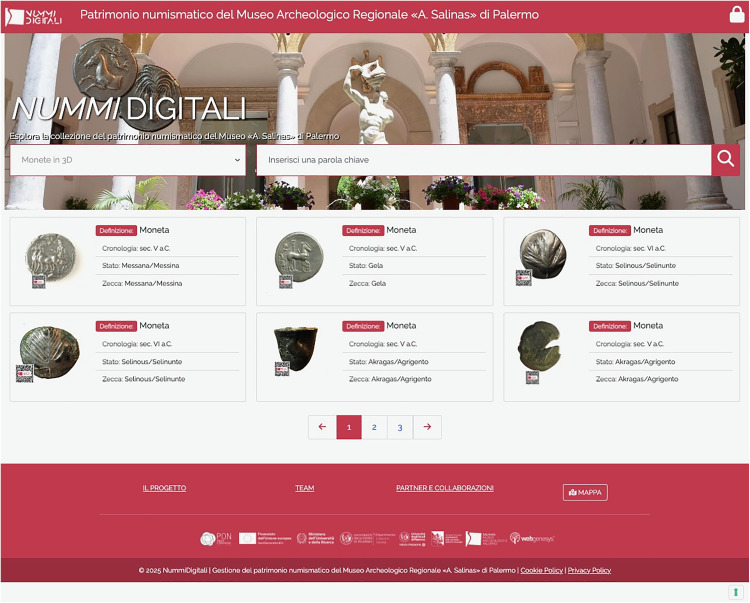
Nummi Digitali website (front-end). Reprinted from https://nummidigitali.it under a CC BY license, with permission from scientific director of the NUMMI DIGITALI, original copyright NUMMI DIGITALI year 2025.

The front-end (available at https://nummidigitali.it) (Fig 2) provides public access to the collection via a web interface that includes:

- simplified data visualization in line with the “public archaeology” [15] principles: a user-friendly version of Scheda NU + , further optimized for broad audiences while maintaining core research fields;
- advanced search tools through filters relating to keywords (definition, nominal, place of discovery, previous collection, inventory number, previous numbers, chronology, obverse, reverse, specifications, engraver’s name, issuers, minters/magistrates, state, mint);
- data request feature: users can obtain full information about the coins and images, through an online request system managed by the Museum;
- interactive high-resolution imaging, with 2D and 3D visualizations;
- geospatial visualization of numismatic production data (mint) and coin finds (findspot of isolated or hoarded specimens);
- security and copyright protection: to prevent unauthorized distribution of images, measures have been implemented, including disabling save, copy, paste, and external page viewing functions, along with a protective QR code overlay containing copyright information.

All data are stored in XML format, ensuring interoperability, data sharing and collaborative research according to the LOD paradigm. Thanks to the adoption of national cataloging standards, “*Nummi* Digitali“ also provides data to SIGECweb, the ICCD operational platform, which guarantees data sharing, in a safe and not obsolescent manner [16]. The alignment process to the nomisma.org ontologies, currently underway, will instead ensure data sharing with the international platforms that use this standard.

### 3D imaging

A core innovation of the “*Nummi* Digitali” platform lies in its capacity to generate high-resolution 3D models of ancient coins with sub-millimeter accuracy [17]. This is achieved through a multimodal digitization pipeline that combines structured-light 3D scanning and macro-photogrammetry to deliver both geometric precision and photorealistic surface rendering (S4 Appendix).

Currently, a variety of imaging and digitization techniques are under active exploration from the scientific community to enhance coin modeling, including structured-light 3D scanning, macro-photogrammetry, and elastomeric-sensor photometric-stereo [18–21].

For 3D acquisition (see Figures C and D in S4 Appendix), we employed the ATOS Q 8M (GOM-ZEISS), a structured-light scanner commonly used in industrial metrology.

The scanner’s key technical specifications include a LED light source; 8 million points per scan; measuring area of 100 x 70 mm to 500 x 370 mm; point spacing ranging from 0.04 to 0.15 mm; working distance of 490 mm.

Its spatial resolution—up to 50 µm—enables the detection of micro-scale iconographic and surface features, including tool marks, traces of overstriking and post-mint alterations. Coins were mounted vertically on a high-precision rotating stage, allowing full circumferential capture via overlapping scans. Obverse and reverse surfaces were digitized in separate sessions and subsequently merged to yield a complete 3D model, accurate to approximately 0.1 mm.

During 3D acquisition, the laser scanner ATOS Q 8M GOM by ZEISS projects a structured pattern of blue light onto the object being scanned, and the deformations in this pattern, caused by the coin’s surface, are analyzed to reconstruct the coin’s 3D shape with high precision. This optical triangulation allows the precise coordinate position of each measured point to be calculated, reconstructing the coin’s shape point-by-point.

To complement geometric data with chromatic fidelity, macro-photogrammetry was employed using a Canon EOS 6D (20 MP) equipped with a 60 mm f/2.8 macro lens. Coins were positioned horizontally on a calibrated rotation plate bearing photogrammetric markers. A total of 32 images per specimen were acquired at 22.5° increments, with additional orthogonal shots for full obverse/reverse documentation. This approach ensured accurate texture mapping and sub-millimetric reproduction of surface coloration and tonality.

### Data processing

**Fig 3:**
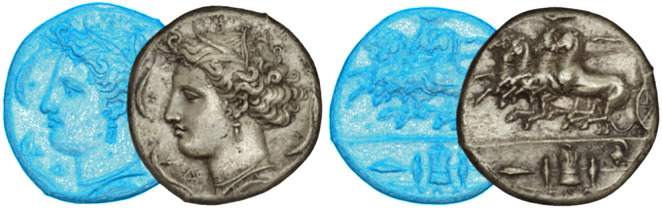
Coin No. 26078. 3D mesh model derived from laser scanning versus textured 3D model derived from photogrammetry.

The integration of laser scanning and photogrammetric data required a multi-step processing pipeline to generate final high-fidelity 3D models of the coins. Raw point clouds from the structured-light scans were processed in GOM Inspect Pro, culminating in the creation of high-resolution polygonal meshes. Independently, the 32 macro-photographs per coin were processed using Agisoft Metashape to reconstruct photogrammetric models with detailed color and texture information. These models were subsequently aligned with the laser-derived meshes through coordinate system matching in Metashape, enabling the seamless transfer of photorealistic textures onto metrically accurate 3D geometries. The result (Fig 3) is a unified model combining sub-millimeter geometric fidelity (up to 0.1 mm) with high-resolution surface textures, preserving fine iconographic details, color variations, and micro-imperfections critical for numismatic analysis (see integrated 3D model in S1 File and S2 File).

### 3D models integration with Sketchfab and database

**Fig 4:**
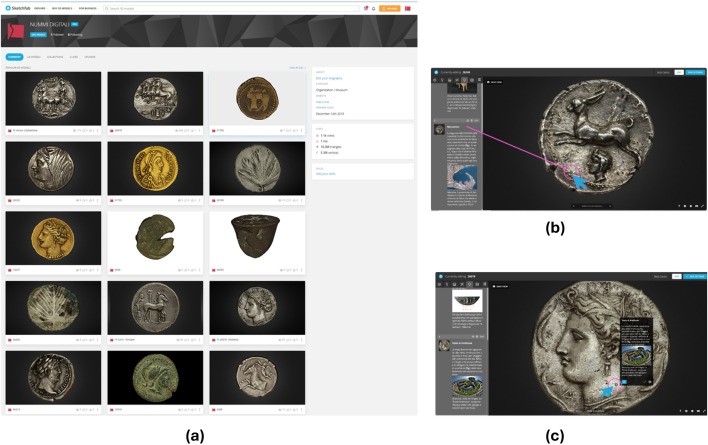
Sketchfab webpage and functions. (a) Models uploaded to the Nummi Digitali profile. (b) Sketchfab annotations: interactive points of interest can be added to the models and information can be accessed by clicking on numbered markers. (c) Model navigation on Sketchfab: clicking on the marker, a window displaying textual information and images related to the point of interest will open.

To enable interactive online visualization of the digitized coins, the final photo-realistic 3D models were exported in .obj format and uploaded to Sketchfab, a web-based platform for high-resolution 3D rendering. A dedicated “*Nummi* Digitali” account was set to host the models (Fig 4). Sketchfab’s interface allows users to freely rotate, zoom, and inspect the coins from multiple perspectives, providing an immersive visual experience. Interactive annotations—such as points of interest linked to hypertextual and multimedia content—were embedded within the models, enhancing user engagement and conveying additional historical and typological information. The Sketchfab viewer was seamlessly integrated into the “*Nummi* Digitali” database, allowing direct access to 3D content within the platform’s virtual museum interface and supporting dynamic exploration across research and educational contexts.

### XRF spectra acquisition

**Fig 5:**
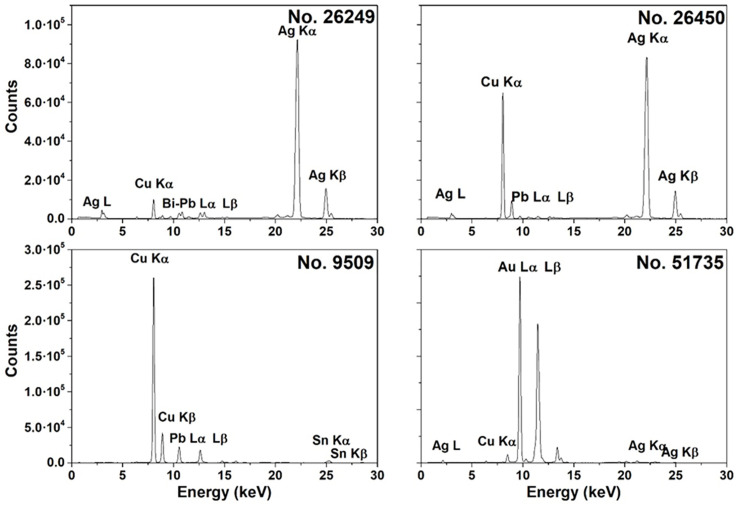
XRF spectra of representative coins in silver (No 26249), copper suberated in silver (No 26450), bronze (No 9509) and gold (No 51735).

The XRF spectra (Fig 5) were acquired in situ using a portable Tracer III SD Bruker AXS spectrometer. The X-ray tube, with a rhodium target, operated at 40 kV and 11 µA, coupled with a 10 mm² silicon drift detector, enabling the detection of elements with atomic number Z > 11.

No vacuum was applied at the instrument’s output, and a filter, of a thin titanium and aluminum layer, was used to eliminate the contribution of lighter elements. For each coin, two XRF spectra were collected one per side, totaling 64 spectra.

The spectral analysis was performed using ARTAX7^®^ software (Version 7.4.6.1) to assign each peak and estimate peak areas, conducting a semi-quantitative analysis based on metallic standards and empirical calibration methods [22,23]. The presence of argon, nickel, palladium, and rhodium signals, attributed to ambient atmosphere and instrumental components, was observed in all spectra, along with the elements composing the coin alloys.

## Results: The impact of “*Nummi* Digitali” project on the “A. Salinas” Archaeological Museum in Palermo

The “*Nummi* Digitali” project has had a significant impact on the study, public enhancement and accessibility of the numismatic collection housed in the “A. Salinas” Regional Archaeological Museum in Palermo. Notably, the museum’s Coin Cabinet, closed to the public for over 70 years, has remained largely inaccessible to scholars and visitors. The digital platform has thus served as a crucial prelude to its reopening, enabling both specialists and the general public to engage with a previously unseen heritage corpus.

Within this context, the integration of 3D models and XRF-based compositional analyses has emerged as a dual-purpose tool: supporting advanced numismatic research while simultaneously enhancing public outreach. The digital surrogates—or “digital twins”—have revealed surface features and manufacturing anomalies not previously documented, offering new directions for numismatic investigation. As demonstrated in the following examples, the combination of metric accuracy and material data has uncovered subtle iconographic reworkings, minting irregularities, and alloy variations, highlighting the potential of the platform to generate novel research questions and foster broader cultural engagement.

In the *Selinous* didrachm N.I. 26169 (510–500 BCE) (see Table C in S5 Appendix) a trace of die reworking on the reverse has emerged. It is the erasure of a letter of the ethnic (SELI) inside the incuse square that encloses the leaf of *selinos*. Although it may seem like a minor change, this alteration may provide some hints about the chronological sequence of the *Selinous* series of the late 6th century BC.

In the *Messana* tetradrachm N.I. 26249 (412–408 BCE) (see Table C in S5 Appendix), the reverse field exhibited an irregularity, which could be attributed to defects in the fabrication of the die or to the practice of overstriking.

In the *Panormos* bronze coin N.I. 10010 (I century BCE), 3D imaging revealed unusually large spacing between iconographic details, raising suspicions about the authenticity of the specimen, further supported by metal composition analysis.

Instead, XRF-based compositional data provided insights into the alloys and on production techniques, highlighting some discrepancies. The spectra were categorized into three groups based on the primary elements detected.

The quantitative analysis on gold and electrum coins (see Table B in S5 Appendix) highlights differences between coinages from different periods, as well as some peculiarities in composition that sometimes appear inconsistent with the socio-political climate of the years in which the specimens were issued.

In the electrum coin N.I. 14537 from Carthage (before 300 BCE), it was expected to contain an alloy of gold and silver [24], while the analysis instead showed an unusually high gold content (92.9%) with only 8% silver. Instead, the solidus N.I. 51735 (402–406 BCE) is composed of nearly pure gold, with a low silver content (0.14%), reaching a fineness of 23.94 carats and attesting to the ability of the Roman Empire to maintain the high intrinsic value of gold coins, despite the war commitments it had to face.

The composition of **silver coins** shows greater variability (see Table C in S5 Appendix). The *Selinous* didrachm N.I. 26450 (540/520–510/500 BCE) is among the earliest coins of the colony and of Sicily. Autoptic examination suggests it is *subaeratus* (a plated coin), as greenish oxidation typical of copper appears on the surface, but metallurgical analysis also confirmed that the coin is composed of silver on the outside and copper in the core.

Another *Selinous* didrachm (N.I. 26169, late 6th century BCE), later than the previous example, exhibits a significant copper content, which however could be attributed to either deliberate addition or incomplete silver refining.

A mixed composition was found in the *Leontinoi* didrachm N.I. 26259 (around 470 BCE) and in this case it may be a specific feature of the alloy used.

In the tetradrachms of Gela N.I. 26117 (465–450 BCE) and *Messana* N.I. 26249 (412–408 BCE)significant amounts of silver along with small traces of lead and bismuth have been detected. These elements may suggest a provenance of the silver ore from Thrace, since recent studies have documented that the silver mines of this region are characterized by such elements [25].

The *Selinous* tetradrachm N.I. 26173 (440–420 BCE) revealed a composition like Punic tetradrachms N.I. 9286 and N.I. 26103 minted between the late 5th and early 4th centuries BCE. The data could suggest a common origin for raw materials, aligning with the historical interactions between Selinuntine and Carthaginian communities. In fact, both the coin minted by Carthage in Sicily (N.I. 9286, 410–390 BCE) and the Punic coin of *Panormos* (N.I. 26103, 410–390 BCE) exhibit comparable compositions (98.0%–99.7% silver, with minimal copper content 1.0%–0.2%, perhaps attributable to impurities in the silver ore), confirming shared techniques and sources of metal supply.

The Syracuse decadrachm N.I. 26078 (late 5th–early 4th century BCE) contains approximately 99% silver, along with small amounts of copper and lead. Remarkably, the same composition characterizes the tetradrachm of Hieron II N.I. 26232 (218/217–214 BCE), minted in Syracuse two centuries later for the king’s wife Philistis.

The denarius of Hadrian N.I. 65413 (130 CE, Rome) is composed of 93% silver and 6% copper, despite the progressive debasement of Roman coinage during the Imperial period, as evidence of the high intrinsic value maintained by the coin in the middle imperial age.

Finally, the composition of **copper coins** (see Table D in S5 Appendix) is equally heterogeneous.

The *Akragas trias* N.I. 64093 (mid-5th century BCE) is made up of a ternary bronze alloy, consisting of copper, lead, and tin: the high lead content is related to the use of the casting technique, which required a more fluid metal.

Conversely, the lead content decreases significantly in the *hemilitron* of the same mint (N.I. 9509, late 5th–early 4th century BCE), since it was produced by minting, rather than casting.

The *Panormos* bronze coin N.I. 10010 (90–50/40 BCE) exhibits lead content beyond the XRF instrument’s calibrated detection limit, making it impossible to determine precise elemental percentages. This anomaly could be due to surface enrichment from environmental exposure or an unusual alloy composition, which is suspicious, and requires further investigation on other coins of the same series. Considering the huge amount of information obtained with XRF results on a limited set of coins, it is planned to perform further analyses on a larger sample to determine whether the results represent isolated cases or broader production trends and to also provide an assessment of minor elements. By combining statistical processing of the data, it will also be possible to highlight differences regarding the basic materials and their provenance [26,27,28].

The integration of numismatics, material science, and computational imaging positions “*Nummi* Digitali” as a transformative tool for scientific research on ancient coins, establishing a replicable framework for future interdisciplinary research in cultural heritage studies.

Currently, “*Nummi* Digitali” establishes the “A. Salinas” Regional Archaeological Museum in Palermo as the first Museum in Italy equipped with an interoperable, web-accessible numismatic database, setting a new benchmark for digital numismatic research and heritage’s enhancement.

## Discussion

The “Nummi Digitali” platform is the result of an innovative approach to the digitization of numismatic heritage, which enhances cross-disciplinary research and public engagement (S6 Appendix).

The system, validated through the pilot study at the “A. Salinas” Regional Archaeological Museum in Palermo, can be easily extended and scaled up to every coin collection. The platform’s architecture is based upon the following pillars:

1. compliance of numismatic cataloging standards with the ICCD “Scheda NU”, with the guidelines [29] of National Digitalization Plan for Cultural Heritage (PND) of the Italian Ministry of Culture and with the FAIR [30] (Findable, Accessible, Interoperable and Reusable) data principles;
2. interoperability and data integration with national and international numismatic repositories, such as SIGECweb and databases aligned to Nomisma.org;
3. implementation of Linked Open Data (LOD) [31], which includes integration with other numismatic databases, facilitating comparative studies across institutions; metadata exchange with numismatic platforms using common or equivalent cataloguing standards; automated linking between coin records and external datasets, enhancing the research accessibility;
4. integration of multidisciplinary data: 1. repository of high-resolution 2D imagery and 3D models reproducing coin surfaces and geometries, obtained via structured-light 3D scanning and macro-photogrammetry; 2. archaeometric data, obtained via non-invasive techniques, such as XRF spectroscopy, for quantitative alloy composition, or invasive, such as lead isotope analysis; 3. contextual archaeological references.

A wide range of advanced 3D modeling methodologies, such as macro-photogrammetry and laser scanning, has been employed and refined for the digitization of ancient coins. These techniques offer precise reconstructions of coins that not only improve visualisation, but also facilitate detailed metric analysis, die studies, the assessment of striking defects, production techniques, and damage, elements that are essential for both research and conservation.

X-ray fluorescence (XRF) spectroscopy provides a non-invasive approach to analyzing the elemental composition of coin alloys and eventual surface treatments, revealing insights into metallurgical techniques, patterns of metal sourcing across different historical periods, and authenticity.

Finally, the comparison between numismatic data and associated archaeological finds within the discovery context represents an indispensable source of information for refining the chronological attribution of coins, as well as for understanding their function and use in antiquity. The database features two complementary interfaces with distinct functions: a back end for data entry, full access, searching and management and a front-end for public visualization and interactive exploration.

The back end is built on the ICCD’s “Scheda NU” [32] cataloging format, which was properly optimized to enhance flexibility, to improve scientific research and accommodate coins of various provenances. This was achieved implementing specific ontologies, validated by the ICCD and by the Institute of Cognitive Sciences and Technologies (ISTC) of the CNR [33]. The revised structure, called “Scheda NU+”, streamlines data collection, enabling guided data entry. Thanks to the adoption of standardized ontologies, platform data, formatted in XML, are interoperable and shareable, even according to the LOD paradigm. The web front-end has been designed to provide a user-friendly interface and seamless connection with the database with advanced capabilities for browsing, querying and requesting data and 3D interactive exploration.

The 3D visualization (via Sketchfab) allows to deepen the knowledge of the artifact for scientific purposes and, at the same time, is a highly attractive element for any user, providing:

- full rotational views, enabling users to examine coins from multiple points of view;
- advanced acquisition of edge and surface details and accurate mapping of the relief articulation, essential for in-depth analysis of the iconography and aspects related to the production of the coin (countermarking, overstriking, defects, retouching, orientation of the dies);
- annotations (points of interest) with hypertextual and multimedia content, providing information about the coin’s history, intrinsic features, and metal composition based on metallurgic analyses.

“*Nummi* Digitali” marks a significant advancement in digital numismatics, as it uses a flexible numismatic cataloging system and it introduces a multi-source dataset enriched by metric models, scientifically validated compositional analyses and contextual archaeological data.

On the one hand, in fact, the updating of cataloging standards has produced a renewed cataloging scheme (Scheda NU+), which has contributed to the streamlining of data collection, broadening the perspective also on contextual discovery data, until now not sufficiently taken into consideration.

On the other hand, 3D digital twins ensure high-quality reproduction, suitable for research as well as for dissemination. Interactive 3D visualizations allow enlargements beyond physical limitations, enabling researchers to observe fine details under variable lighting conditions. This approach supports attributions and facilitates the study of images and production techniques (die reworkings, overstriking) and, when necessary, the identification of counterfeit or altered specimens, particularly relevant for historical collections formed through donations or acquisitions.

Furthermore, the XRF spectroscopic analysis provides valuable information regarding the elemental composition, to better define the coin from a metallurgical point of view. The integration of XRF spectroscopy results into the coin data records and annotations of the 3D models further enhances the value of the platform, also as an educational tool, beyond research.

Finally, the interoperability with existing repositories enhances comparative numismatic studies, while the possibility of replicability of the platform makes “Nummi Digitali” a scalable tool for the global numismatic community.

In addition to its impact on academic research, the open-access nature of the platform allows museums, curators, and independent scholars to explore numismatic collections in an interactive and scientific way, bridging the gap between specialist research and public engagement.

## Conclusion

The *Nummi Digitali* project has established a pioneering digital ecosystem for the interdisciplinary study, cataloging, and dissemination of ancient coins. Through the integration of structured cataloging protocols, high-resolution 3D models, and non-invasive archaeometric data, the platform has redefined the methodological standards of digital numismatics. Its implementation at the “A. Salinas” Regional Archaeological Museum has demonstrated the effectiveness of this multimodal approach in enhancing both research capabilities and public accessibility.

Building upon this foundation, the ongoing evolution of the platform is focused on extending interoperability and aligning Italian cataloging standards with the ontologies developed by the Nomisma.org network. This semantic convergence will enable data integration across national and international repositories, supporting collaborative research on a global scale and ensuring the long-term sustainability and reusability of numismatic datasets in accordance with FAIR and LOD principles. The renewed platform will also introduce:

- **Multilingual localization**, enabling the translation of both field labels and contents.
- **Alignment of archaeometric modules**, ensuring uniformity in how physical-chemical analyses (e.g., XRF, isotopic data) are integrated and visualized within catalog records.
- **Advanced filtering tools** for textual and visual queries, allowing users to search by hoard association, availability of 3D models, and to sort records chronologically or by typological categories.
- **Geospatial functionalities**, integrating geolocation metadata for findspots and mint locations, thus enhancing the archaeological contextualization of each coin.
- **Optimization of unique identifiers**, ensuring unambiguous, scalable, and persistent referencing of each digital object across systems.

Future expansions will also explore the integration of artificial intelligence (AI) and machine learning (ML) to address challenges at two progressive levels of knowledge extraction and automation.

1. AI-assisted querying: simple information retrieval tasks can be performed via AI tools using natural language prompts to query structured metadata in the database. For example, the system could generate dynamic lists of coins sharing specific features—such as issuing authority, typology, iconography, or metal composition—based on user-defined parameters. This functionality would enhance user interaction, especially for non-specialists, by transforming complex database queries into intuitive semantic requests.
2. Semi-automatic classification and anomaly detection: Machine learning models, particularly convolutional neural networks (CNNs), can be trained on the high-resolution visual and geometric data (2D images and 3D meshes) linked to “Scheda NU+” records. By correlating objective parameters such as weight, diameter, morphology, and iconographic details, these models can assist in the identification of coin typologies, detect die-linked specimens, and flag potential anomalies or stylistic inconsistencies.

CNNs have already demonstrated efficacy in landmark recognition on coins [34], significantly improving classification accuracy by identifying distinctive features with precision. Within the *Nummi* Digitali architecture, these AI functionalities could be embedded into the back-end system to provide predictive suggestions during cataloging or to support expert verification processes. Operating in synergy with structured metadata and interoperable ontologies, this integration would not only enhance scalability and efficiency but also foster the development of advanced computational numismatics and interdisciplinary research tools.

Ultimately, the platform’s open-access model, modular design, and interdisciplinary framework render it a replicable solution for cultural institutions managing numismatic collections. It exemplifies how digital infrastructures can bridge disciplinary divides, support multilevel access, and ensure the preservation and valorization of numismatic heritage in both academic and public domains.

## Supporting information

S1 Appendix Numismatic Databases. The file presents a comparative overview of major online numismatic databases, establishing the digital context for the project. It outlines the diversity of platforms, cataloging standards, and technological approaches adopted by institutions across Europe. By mapping the existing digital landscape, this section defines the motivations behind the development of the *Nummi Digitali* platform and highlights the project’s contribution to overcoming current limitations in data integration, 3D accessibility, and archaeometric interoperability.
(PDF)

S2 Appendix Selection of coins from the “A. **Salinas” Regional Archaeological Museum of Palermo**. This section outlines materials and methods, including the selection criteria and analytical data for 15 coins from the “A. Salinas” Regional Archaeological Museum of Palermo (Table S1), a technical description of the platform architecture (with S1–S2 Figs), and the integrated 3D acquisition workflow for coin digitization (S3 Fig).
(PDF)

S3 Appendix Platform architecture. This file details the structure and metadata logic of the Nummi Digitali platform, composed of a back-end (for cataloging and expert queries) and a front-end (for public access and visualization). The system is built on the ICCD “Scheda NU+” standard, and integrates distinct metadata sections with vocabulary-guided input. Supplementary S1 and S2 Figs illustrate the interface components and cataloging structure.
(PDF)

S4 Appendix 3D Imaging. This section describes the methodologies used for digital acquisition, including photogrammetry and structured-light 3D laser scanning, as applied to ancient coins. It discusses instrumentation, setups, resolution strategies, and limitations, with reference to recent literature and comparative approaches. Supplementary S3 Fig documents both scanning systems used: (a) laser scanning with ATOS Q and GOM ROT 350; (b) macro-photogrammetry with Canon EOS 7D and Leica lens.
(PDF)

S5 Appendix XRF data. This file presents the full results of XRF analyses performed on the selected coins, organized in three tables. Table S2 reports Au, Ag, Cu concentrations and carat values for gold and electrum coins; Table S3 shows Ag and Cu content in silver coins; Table S4 details multi-elemental data for copper-based specimens. Anomalies due to subaeration or high Pb content are noted where applicable.
(PDF)

S6 Appendix Results. This section summarizes the key innovations and results of the *Nummi* Digitali pilot, including the implementation of a SaaS-based infrastructure, integration of ICCD-compliant metadata with 3D and analytical content, and the digitization of a representative numismatic corpus.
(PDF)

S7 Appendix Author Contributions. This file details the specific roles of each author in drafting selected sections of the main article and the Supporting Information
(PDF)

S1 File Coin No. 26078. Integrated 3D model of the coin in 3D format.
(ZIP)

S2 File Coin No. 26269. Integrated 3D model of the coin in 3D format.
(ZIP)

## References

[1] Nomisma.org. Accessed 2025 Apr 23. http://nomisma.org

[2] Meadows A, Gruber E. Coinage and numismatic methods: A case study of linking a discipline. ISAW Pap. 2014. Accessed 2025 Jul 28. http://dlib.nyu.edu/awdl/isaw/isaw-papers/7/meadows-gruber

[3] Gruber E, Meadows A. Numismatics and linked open data. In: Bond SE, Dilley P, Horne R, editors. Linked Open Data for the Ancient Mediterranean: Structures, Practices, Prospects. ISAW Pap; 2021.

[4] Istituto Centrale per il Catalogo e la Documentazione ICCD. Standard catalografici. Accessed 2024 Oct 28. http://www.iccd.beniculturali.it/it/standard-catalografici

[5] ICCD – MiBACT. Standard catalografici repository. Accessed 2025 Jul 16. https://github.com/ICCD-MiBACT/Standard-catalografici.git

[6] ICCD – Mibact, Scheda NU. Accessed 2025 Jul 16. http://iccd.beniculturali.it/getFile.php?id=155

[7] Gandolfo L. Il medagliere del museo “Antonino Salinas”. Compte Rendu INC. 2014;61:49–53.

[8] Sole L. La collezione numismatica greca e romana del Museo Archeologico Regionale “Antonino Salinas” di Palermo: studi e ricerche. In: Parello MC, editor. L’isola dei tesori. Ricerca archeologica e nuove acquisizioni. Bologna: [editore, se noto]; 2024. p. 39–47.

[9] Arslan E, Bianchin Citton E, Callegher B, Ferrante F, Giovetti P, Mancinelli ML. Strutturazione dei dati delle schede di catalogo. Roma; 2004.

[10] Nomisma.org. Numismatic Description Scheme NuDS. Accessed 2025 Apr 23. http://nomisma.org/nuds

[11] Mancinelli ML, Veninata C. Architettura della conoscenza: il Sistema ICCD come modello per la descrizione dei beni culturali. In: Mieli M, Volpe C, editors. Conferenza GARR 2018 Selected Papers. Roma. 2019. p. 130–4.

[12] Mancinelli ML, Veninata C. Patrimonio numismatico e catalogo generale dei beni culturali: progetti in corso per l’integrazione e la valorizzazione delle conoscenze. In: Pennestrì S, editor. Verso il futuro: Esperienze, progetti e casi di studio tra tutela, fruizione e comunicazione del patrimonio numismatico pubblico. Notiziario Portale Numism. 2022. p. 479.

[13] Mancinelli ML. Normativa trasversale. Versione 4.00. Strutturazione dei dati e norme di compilazione. Roma. 2017.

[14] Sole L. Nummi Digitali: approcci innovativi per la conoscenza, gestione e valorizzazione del patrimonio numismatico del Museo Archeologico Regionale A. Salinas di Palermo. In: Pennestrì S, editor. Verso il futuro: Esperienze, progetti e casi di studio tra tutela, fruizione e comunicazione del patrimonio numismatico pubblico. Notiziario Portale Numism; 2022. p. 539–47.

[15] Volpe G. Archeologia pubblica: metodi, tecniche, esperienze. Roma. 2020.

[16] Birozzi C, Barbaro B, Mancinelli ML, Negri A, Plances E, Veninata C. Catalogare nel 2020. La digitalizzazione del patrimonio culturale. Aedon. 2020. Accessed 2025 Apr 23. http://www.aedon.mulino.it/archivio/2020/3/birrozzi.htm

[17] Zambanini S, Schlapke M, Hödlmoser M, Kampel M. 3D acquisition of historical coins and its application area in numismatics. In: Stork D, Coddington J, Bentkowska-Kafel A, editors. Computer Vision and Image Analysis of Art. SPIE & IS&T – The Society for Imaging Science and Technology; 2010. doi: 10.1117/12.840203

[18] Ponterio RC, Castrizio D, Renda V, Giuffrida D. Applicazioni di micro-profilometria laser e modellazione 3D per lo studio di due reperti numismatici provenienti da Reggio Calabria. In: Puglisi M, Mondello C, editors. Acts of “The 8th Joint Meeting of ECFN and nomisma.org on Coin Finds and Digital Numismatics”. Messina: Messina University Press; 2024. doi: 10.13129/979-12-80899-12-5

[19] Castellani U, Bartolomioli R, Marchioro G, Calomino D. From coin to 3D face sculpture portraits in the round of Roman emperors. Computers & Graphics. 2024;123:103999. doi: 10.1016/j.cag.2024.103999

[20] Calomino D. Digital casts and 3D models: new ways of looking at Roman Emperors on coins. L’Antiquité à la BnF. 2025 Jun 17. 10.58079/14560

[21] Calomino D, Bologna F, Wilson FP, Donnelly M, Williams MA. Imaging Hadrian in Britain between coinage and sculpture: a new digital approach to the study of Roman imperial portraiture. Britannia. 2023;54:251–74.

[22] Caponetti E, Armetta F, Chillura Martino D, Saladino ML, Ridolfi S, Chirco G. First discovery of orichalcum ingots from the remains of a 6th century BC shipwreck near Gela Sicily. Mediterr Archaeol Archaeom. 2017;11. doi: 10.5281/zenodo.581716

[23] Armetta F, Nardo VM, Trusso S, Saladino ML, Arcovito A, Cosio E, et al. The silver collection of San Gennaro treasure (Naples): A multivariate statistic approach applied to X-ray fluorescence data. Spectrochimica Acta Part B: Atomic Spectroscopy. 2021;180:106171. doi: 10.1016/j.sab.2021.106171

[24] Jenkins GK, Lewis RB. Carthaginian gold and electrum coins. Royal Numism Soc Spec Publ. 1963;2:92.

[25] L’Héritier M, Baron S, Cassayre L, Téreygeol F. Bismuth behaviour during ancient processes of silver–lead production. Journal of Archaeological Science. 2015;57:56–68. doi: 10.1016/j.jas.2015.02.002

[26] Armetta F, Nardo VM, Trusso S, Saladino ML, Arcovito A, Cosio E, et al. The silver collection of San Gennaro treasure (Neaples): A multivariate statistic approach applied to X-ray fluorescence data. Spectrochimica Acta Part B: Atomic Spectroscopy. 2021;180:106171. doi: 10.1016/j.sab.2021.106171

[27] Caponetti E, Armetta F, Brusca L, Chillura Martino D, Saladino ML, Ridolfi S, et al. A multivariate approach to the study of orichalcum ingots from the underwater Gela’s archaeological site. Microchemical Journal. 2017;135:163–70. doi: 10.1016/j.microc.2017.09.003

[28] Caponetti E, Armetta F, Brusca L, Ferrante M, Chillura Martino D, Saladino ML, et al. Newly discovered orichalcum ingots from Mediterranean sea: Further investigation. Journal of Archaeological Science: Reports. 2021;37:102901. doi: 10.1016/j.jasrep.2021.102901

[29] Mancinelli L. Gli standard catalografici dell’Istituto Centrale per il Catalogo e la Documentazione. In: Tucci R, editor. Le voci, le opere e le cose. La catalogazione dei beni culturali demoetnoantropologici. Roma; 2018. p. 279–302.

[30] Wilkinson MD, Dumontier M, Aalbersberg IJJ, Appleton G, Axton M, Baak A, et al. The FAIR Guiding Principles for scientific data management and stewardship. Sci Data. 2016;3:160018. doi: 10.1038/sdata.2016.18 26978244 PMC4792175

[31] World Wide Web Consortium W3C. Linked Data. Accessed 2025 Apr 27. https://www.w3.org/DesignIssues/LinkedData

[32] Istituto Centrale per il Catalogo e la Documentazione ICCD. Scheda NU. Beni numismatici. Accessed 2025 Apr 23. http://www.iccd.beniculturali.it/it/ricercanormative/18/nu-beni-numismatici-3_00

[33] Mancinelli ML, Veninata C. Patrimonio numismatico e catalogo generale dei beni culturali: progetti in corso per l’integrazione e la valorizzazione delle conoscenze. In: Pennestrì S, editor. Verso il futuro: Esperienze, progetti e casi di studio tra tutela, fruizione e comunicazione del patrimonio numismatico pubblico. Notiziario del Portale Numismatico dello Stato. 2022. p. 479.

[34] Kim J, Pavlovic V. Discovering characteristic landmarks on ancient coins using convolutional networks. J Electron Imaging. 2016;26(1):011018. doi: 10.1117/1.jei.26.1.011018

