# Correction: *Nummi* Digitali: A pioneering multimodal platform for numismatic heritage

**DOI:** 10.1371/journal.pone.0340618

**Published:** 2026-01-06

**Authors:** Lavinia Sole, Dario Giuffrida, Elisa Chiara Portale, Francesco Armetta, Massimiliano Perri, Oreste Adinolfi, Maria Luisa Saladino, Rosina Celeste Ponterio

There are errors in the Acknowledgement statement. The correct Acknowledgement statement is as follows: The authors (S7 Appendix) thank the project partners for their valuable support.: Regional Archaeological Museum “A. Salinas” in Palermo (Giuseppe Parello, Director of Salinas Museum; Elena Pezzini, archaeologist of Salinas Museum); Web¬genesys S.p.A.; Istituto Centrale per il Catalogo e la Documentazione of the italian Ministero della Cultura. We are grateful for the collaboration also with the Centro Regionale Inventario, Catalogazione e Documentazione (CRICD) of the Sicily Region (https://cricd.it/). D.G. thanks European Union (NextGeneration EU), through the MUR-PNRR project SAMOTHRACE—Sicilian Micro and Nano Technology Research and Innovation Center (ECS00000022). F.A. thanks the Eurostart Project “Armi puniche o greche? la risposta nell’invisibile,” through the “MUR PNR D.M. 737/2021” CUP: B79J21038330001. L.S. thanks European Union (NextGeneration EU), through the MUR D.M. 737/2021 project NUMMI DIGITALI towards Europe (NUDItEu) (CUP B79J21038330001). MLS and FA thank the Project MML-ARCH - “Metodologie di machine learning applicate all’archeometria: una nuova frontiera per l’interpretazione materica dei Beni Culturali”, Programma “CHANGES Cultural Heritage Active iNnovation for Sustainable Society” CUP B53C22003890006 – Codice Identificativo PE_00000020, finanziato dall’Unione Europea – Next Generation EU sui fondi PNRR MUR – M4C2 – Investimento 1.3 “Partenariati estesi a Università, centri di ricerca, imprese e finanziamento progetti di ricerca.

There are also errors in the Funding statement. The correct Funding statement is as follows: D.G. thanks European Union (NextGeneration EU), through the MUR-PNRR project SAMOTHRACE—Sicilian Micro and Nano Technology Research and Innovation Center (ECS00000022). F.A. thanks the Eurostart Project “Armi puniche o greche? la risposta nell’invisibile,” through the “MUR PNR D.M. 737/2021” CUP: B79J21038330001. L.S. thanks European Union (NextGeneration EU), through the MUR D.M. 737/2021 project NUMMI DIGITALI towards Europe (NUDItEu) (CUP B79J21038330001). MLS and FA thank the Project MML-ARCH - “Metodologie di machine learning applicate all’archeometria: una nuova frontiera per l’interpretazione materica dei Beni Culturali”, Programma “CHANGES Cultural Heritage Active iNnovation for Sustainable Society” CUP B53C22003890006 – Codice Identificativo PE_00000020, finanziato dall’Unione Europea – Next Generation EU sui fondi PNRR MUR – M4C2 – Investimento 1.3 “Partenariati estesi a Università, centri di ricerca, imprese e finanziamento progetti di ricerca.
