# Supplementary material for: Nummi Digitali: A pioneering multimodal platform for numismatic heritage
Source: PLoS One. 2025 Oct 3;20(10):e0332151. doi: 10.1371/journal.pone.0332151 (PMC12494253; doi:10.1371/journal.pone.0332151)
Supplement: S1 Appendix — The file presents a comparative overview of major online numismatic databases, establishing the digital context for the project. It outlines the diversity of platforms, cataloging standards, and technological approaches adopted by institutions across Europe. By mapping the existing digital landscape, this section defines the motivations behind the development of the Nummi Digitali platform and highlights the project’s contribution to overcoming current limitations in data integration, 3D accessibility, and archaeometric interoperability. (PDF) [file pone.0332151.s001.pdf]

S1 Appendix. Numismatic Databases

The rapid digital transformation that has swept through our communities in recent decades has had a marked impact on numismatic research. In recent years, cultural and academic institutions have promoted major initiatives aimed at developing thematic numismatic projects and digitising significant coin collections or assemblages. These efforts are consistently directed towards the creation of online-accessible databases and the dissemination of a shared corpus of numismatic evidence, the knowledge and/or accessibility of which is essential to the advancement of scientific research.

Among recent initiatives are online databases dedicated to cataloguing coin finds from archaeological contexts. In Italy, *Coin Finds Hub Italy* (https://www.coinfindshub.it/it/), currently under development by the University of Salerno, aims to document numismatic artifacts from archaeological contexts throughout the national territory. This platform will also host *Dip_Coins – Digital Paestum Coins*, focused on coin finds from the Archaeological Park of Paestum. The *Nomismata* database (https://byzantine.units.it/), curated by the University of Trieste, provides a geo-chronologically indexed archive of Byzantine coins dated between 498 and 1453 CE.

In the broader Mediterranean context, the *Online Greek Coinage* project (https://www.greekcoinage.org/) offers a comprehensive database of Greek coin issues with linked exemplars in major public collections. The *Moneda Iberica* platform (https://monedaiberica.org/?lang=lg-ita) focuses on coinages of the Iberian Peninsula and southern France, while *Pella* (https://numismatics.org/pella/?lang=it) is dedicated to the coinage of Macedonia. The *Ptolemaic Coins Online* initiative (https://numismatics.org/pco/) gives access to Ptolemaic issues minted in Egypt, and the *Corpus Nummorum Online* (https://www.corpus-nummorum.eu/) covers Moesia, Thrace, Mysia, and the Troad.

Roman coinages are represented in *Coinage of the Roman Republic Online* (https://numismatics.org/crro/), which digitizes Michael Crawford's *Roman Republican Coinage*, and in *Online Coins of the Roman Empire* (https://numismatics.org/ocre/?lang=it), which spans from Augustus to Zeno. The *Roman Provincial Coinage Online* project (https://rpc.ashmus.ox.ac.uk/) gathers provincial issues across the Empire, incorporating volumes published since 1992.

Databases dedicated to coin hoards include the *Inventory of Greek Coin Hoards* (http://coinhoards.org/), *Coin Hoards of the Roman Republic* (http://numismatics.org/chrr/), and *Coin Hoards of the Roman Empire* (http://chre.ashmus.ox.ac.uk/), which offer systematic documentation of hoard finds.

Several platforms also focus on institutional collections. Italy's *Vetrine Virtuali* (https://www.medaglieri.numismaticadellostato.it/#/vetrine-virtuali/lista-espositori), coordinated by the Ministry of Culture, consolidates numismatic holdings from museums

across the peninsula. Comparable efforts exist in Austria (e.g., https://www.ikmk.at/, http://gams.uni-graz.at/numis, https://www.univie.ac.at/ikmk), Belgium (https://exploratorium.galloromeinsmuseum.be/, https://www.kbr.be/), France (http://bnf.fr/), Ireland (http://dx.doi.org/10.7925/drs1.ucdclm_10), the Netherlands (https://nnc.dnb.nl/dnb-nnc-ontsluiting-frontend/#/collectie/), the United Kingdom (https://www.britishmuseum.org/, https://library.leeds.ac.uk/, https://hcr.ashmus.ox.ac.uk/, http://www.fitzmuseum.cam.ac.uk/), Spain (https://www.mupreva.org/nomisma/data, https://ceres.mcu.es), and Switzerland (https://ikmk-win.ch/, https://www.nationalmuseum.ch/).

Germany, notably, hosts the most extensive network of digital numismatic resources, with platforms developed by institutions such as the Johannes Gutenberg-Universität Mainz (https://numid.uni-mainz.de/), the Münzkabinett Berlin (https://ikmk.smb.museum/), and over twenty additional university and museum initiatives (e.g., https://ikmk.uni-freiburg.de/, https://ikmk.uni-trier.de/, https://numid.uni-koeln.de/).

Collectively, these projects demonstrate the potential of open and linked data in advancing research and enabling integrated analyses across collections. However, their scope and technological approaches remain heterogeneous, particularly in terms of standardization, 3D imaging, and integration with archaeometric data. In this regard, platforms like *Nummi Digitali* propose a novel, multimodal solution, addressing these limitations and fostering a unified digital ecosystem for numismatic research.
