# Supplementary material for: Nummi Digitali: A pioneering multimodal platform for numismatic heritage
Source: PLoS One. 2025 Oct 3;20(10):e0332151. doi: 10.1371/journal.pone.0332151 (PMC12494253; doi:10.1371/journal.pone.0332151)
Supplement: S2 Appendix — Salinas” Regional Archaeological Museum of Palermo. This section outlines materials and methods, including the selection criteria and analytical data for 15 coins from the “A. Salinas” Regional Archaeological Museum of Palermo (Table S1), a technical description of the platform architecture (with S1–S2 Figs), and the integrated 3D acquisition workflow for coin digitization (S3 Fig). (PDF) [file pone.0332151.s002.pdf]

S2 Appendix. Selection of coins from the "A. Salinas" Regional Archaeological Museum of Palermo.

The list of the selected coins is reported in Table A.

Table A: List of the selected coins

Inv.	References	URL "*Nummi* Digitali" website (https://nummidigitali.it)	Summary of chemical analysis data (for further details, see III.1. XRF data, Table S2)
No. 26450	Macaluso 2008, p. 89, n. 21. (1)<br>Cfr. Arnold-Biucchi 1992, p. 16, n. 6. (2)	https://nummidigitali.it/public/scheda.php?s=9f97376941692616bad91935599c0cfa	The calculation of concentration of Ag and Cu was not possible because it is a *subaeratus* exemplar.
No. 26169	Macaluso 2008, p. 75, n. 18. **(1)**<br>Cfr.: Arnold-Biucchi 1992, p. 17, n. 9 **(2)**	https://nummidigitali.it/public/index.php?campo=INVN&keyword=26169	Ag 95.3±0.8<br>Cu 3.7±0.7
No. 26259	Gandolfo 1990, p. 372, n. 200. (3)<br>Cfr.: SNG Cop. Italy-Sicily, I, 339. (4)	https://nummidigitali.it/public/scheda.php?s=592473f22dd880126e17f12e15cbf7ea	Ag 98.6±0.1<br>Cu 1.2±0.1
No. 26117	Macaluso 1990, p. 364, n. 183. (5)<br>Cfr.: Jenkins 1970, p. 228, n. 240. (6)	https://nummidigitali.it/public/scheda.php?s=6446057158b043c4d1269af1af0fb4dc	Ag 99.58±0.06<br>Cu 0.21±0.03
No. 64093	Cfr.: Westermark 2018, pp. 160–161, Series B, n. 525, A, nn. 10-57 (7)	https://nummidigitali.it/public/scheda.php?s=ce58351aa30bdf30c16d663720f0516a	Fe 0.07±0.03<br>Cu 67±3<br>Zn 0.05±0.02<br>As 1.5±0.1<br>Pb 24±3<br>Bi 0.12±0.01

			Sn 5.2±0.6<br>Sb 0.13±0.05
No. 26173	Macaluso 1990, p. 377, n. 210. **([8])**<br>Cfr.: Schwabacher 1925, p. 7, n. 4. **([9])**	https://nummidigitali.it/public/index.php?campo=INVN&keyword=26173	Ag 99.1±0.2<br>Cu 0.6±0.2
No. 26249	Cfr.: Caccamo Caltabiano 1993, p. 297, n. 606, tav. 36. **([10])**	https://nummidigitali.it/public/scheda.php?s=4628894d9e36dc5cd71fff1081dae382	Ag 97± 1<br>Cu 1.8±0.2
No. 9509	Bibl.: Gabrici 1927, p. 115, n. 42. **([11])**	https://nummidigitali.it/public/scheda.php?s=4ed161f80f7e3fc01bd278db0eb4c839	Fe 0.24±0.06<br>Cu 73±5<br>Zn 0.09±0.01<br>As 1.4±0.4<br>Pb 15±7<br>Bi 0.6±0.2<br>Sn 7±1<br>Sb 0.23±0.03
No. 26103	Bibl.:<br>Salinas 1888, p. 301, n. 73. **([12])**<br>Cfr.: Jenkins 1971, p. 47, n. 29. **([13])**	https://nummidigitali.it/public/scheda.php?s=1cc44abb2e5fe6bf8a7d7c699d4c1d11	Ag 99.67±0.05<br>Cu 0.15±0.05
No. 9286	Bibl.: Jenkins 1974, p. 38, n. 33, series 1. **([14])**	https://nummidigitali.it/public/scheda.php?s=9ad113e695172f8bad63b87de7d23374	Ag 98±1<br>Cu 1.0±0.5
No. 26078	Bibl.: Salinas 1888, p. 300, n. 55. **(12)** Cfr.: Gallatin 1930, 26-27, D II-RIX. 12. **([15])**	https://nummidigitali.it/public/scheda.php?s=b552b669732c6366f0a9b3245f98c0d4	Ag 99.2±0.3<br>Cu 0.5±0.3
No. 14537	Cfr.:Jenkins–Lewis 1963, p. 92, no. 189.1. ([16])	https://nummidigitali.it/public/scheda.php?s=5e4c629eebc37338b9ea83ce035594c8	Ag 8 ± 1<br>Au 92.9 ± 0.9<br>Cu 0.2 ± 0.1
No. 26232	Cfr.: Caccamo Caltabiano, Carroccio, Oteri 1997, p. 178, n. 206, tav. XX. ([17])	https://nummidigitali.it/public/scheda.php?s=0ba9e0cd2f0e59ce640105bc9f21de4f	Ag 99.41 ± 0.08<br>Cu 0.1 ± 0.1
No. 10010	Bibl.: Gabrici 1927, p. 161, n. 292, pl.	https://nummidigitali.it/public/scheda.php?	Coin with high lead content

	VIII, 2. (11)	s=49f117223bb7d6f8a4419d7b59985d19	(over 40%)
No. 65413	Cfr.: RIC II.3, n. 1400. ([18])	https://nummidigitali.it/public/scheda.php?s=d22432d9ee274f2f445db67c8bbf55ff	Ag 93.0±0.6<br>Cu 6.2±0.6
No. 51735	Cfr. RIC X, 1287 ([19])	https://nummidigitali.it/public/scheda.php?s=44a0710b6e15d6dfa70580a32dce3c7f	Ag 0.14 ± 0.01<br>Au 99.74 ± 0.03

References

[1] Macaluso R. La Sicilia e la moneta. Dai mezzi di scambio premonetari alla coniazione in argento dell'unità ponderale indigena. Suppl Kokalos. 2008;20.

[2] Arnold-Biucchi C**.** The beginnings of coinage in the West: Archaic Selinous. In: Nilsson H, editor. *Florilegium numismaticum. Studia in honorem U. Westermark edita*. Stockholm; 1992. pp. 13–19.

[3] Gandolfo L**.** Scheda n. 200. In: *Lo stile severo in Sicilia. Dall'apogeo della tirannide alla prima democrazia*. Palermo; 1990. p. 372.

[4] Sylloge Nummorum Graecorum. The Royal Collection of Coins and Medals, Copenhagen**,** Danish National Museum, I: Italy–Sicily. West Milford (NJ): Classical Numismatic Group; 1981.

[5] Macaluso R. Scheda n. 183. In: *Lo stile severo in Sicilia. Catalogo della Mostra*. Palermo: Museo Archeologico Regionale; 1990. p. 364.

[6] Jenkins GK. The coinage of Gela. In: *AmuGS II*. Berlin; 1970.

[7] Westermark U**.** The coinage of Akragas c. 510–406 BC. Vols. I–II. Stud Numism Upsaliensia. 2018;6(1).

[8] Macaluso R. Scheda n. 210. In: *Lo stile severo in Sicilia. Dall'apogeo della tirannide alla prima democrazia*. Palermo; 1990. p. 377.

[9] Schwabacher W. Die Tetradrachmenprägung von Selinunt. MBNG. 1925;43.

[10] Caccamo Caltabiano M. La monetazione di Messana con le emissioni di Rhegion dell'età della tirannide. Berlin–New York: de Gruyter; 1993.

[11] Gabrici E. La monetazione del bronzo nella Sicilia antica. Palermo; 1927.

[12] A. Salinas, Ripostiglio siciliano di monete antiche d'argento. NSc (1888), pp. 295–312.

[13] Jenkins GK. Coins of Punic Sicily. Part 1. Schweizer Numism Rundsch. 1971;50:1–78.

[14] Jenkins GK. Coins of Punic Sicily. Part 2. Schweizer Numism Rundsch. 1974;53:23–41.

[15] Gallatin A. Syracusan dekadrachms of the Euainetos type. Cambridge: Harvard University Press; 1930.

[16] G. K. Jenkins, R. B. Lewis, Carthaginian Gold and Electrum Coins (London, 1963).

[17] Caccamo Caltabiano M, Carroccio B, Oteri E. Siracusa ellenistica. Le monete 'regali' di Ierone II, della sua famiglia e dei Siracusani. Pelorias. 1997;2.

[18] Abdy RA, Mittag PF, editors. *Roman Imperial Coinage II.3: From AD 117 to AD 138 – Hadrian*. London: Spink; 2019..

[19] Kent JPC, Carson RAG. *Roman Imperial Coinage, Vol. X*. London: Spink; 1994.
