# Supplementary material for: Nummi Digitali: A pioneering multimodal platform for numismatic heritage
Source: PLoS One. 2025 Oct 3;20(10):e0332151. doi: 10.1371/journal.pone.0332151 (PMC12494253; doi:10.1371/journal.pone.0332151)
Supplement: S3 Appendix — This file details the structure and metadata logic of the Nummi Digitali platform, composed of a back-end (for cataloging and expert queries) and a front-end (for public access and visualization). The system is built on the ICCD “Scheda NU+” standard, and integrates distinct metadata sections with vocabulary-guided input. Supplementary S1 and S2 Figs illustrate the interface components and cataloging structure. (PDF) [file pone.0332151.s003.pdf]

S3 Appendix. Platform architecture.

The *Nummi* Digitali platform is structured into two integrated components: a back end for data entry, cataloging, and expert search, and a front end for public access and interactive visualization. **Supplementary figure 1** provides a schematic overview of this architecture, showing the data flow from acquisition and processing to user interaction, enabling multilevel access and interoperability.

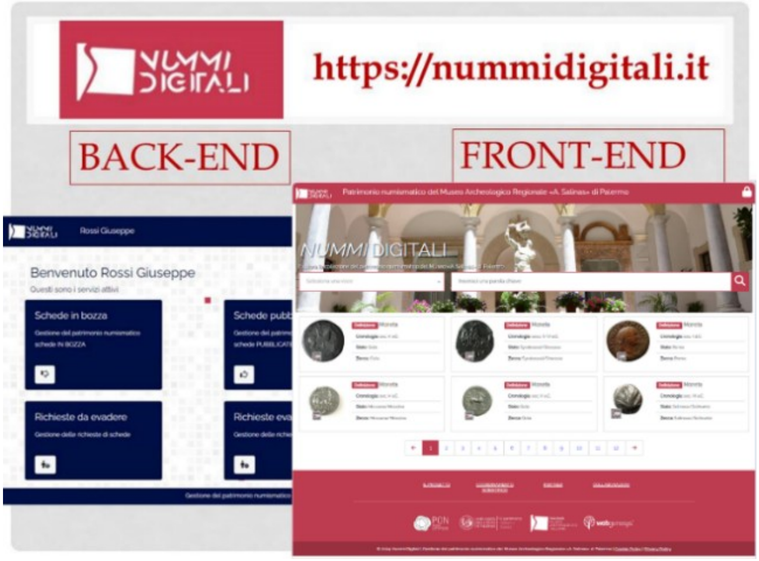

Figure A: “*NUMMI* DIGITALI” platform: the two interfaces (the back-end and the front-end).

The **back-end** interface is based on the “Scheda NU+”, that comprises 19 sections (**Fig. S2**):

- Identification codes: it collects unique identification numbers and letters for each coin (**Fig. S2a**).
- Object description: it details the type of object (e.g., coin, token), denomination, series, classification, availability, and specific features such as imitations or overstriking (**Fig. S2b**).
- Administrative data (**Fig. S2c**): it includes information on the museum, storage location, inventory number, economic value, acquisition details, and legal status.
- Historical locations (**Fig. S2d**): it documents the previous locations of the coins, whether from collections or archaeological finds, with topographic details and georeferencing links.
- Archaeological context: it provides detailed excavation data (**Fig. S2e**), including discovery area, nature of discovery, stratigraphic units (**Fig. S2f**), and contextual materials.
- Chronology: it records the general or specific dating of the coins.
- Technical Data: it includes physical measurements and technical specifications.
- Analytical Data: it covers descriptions, inscriptions, issuing authorities, mints and countermarks, with fields for overstriking and testing.
- Conservation and restoration: it notes the condition, and any restoration work or analysis performed on the coins.
- References and documentation: it allows for the uploading of images, bibliographic references and other relevant documents.
- Data access: specifies the accessibility of the data.
- Compilation: records of the data entry personnel and museum officials.

Each section can be expanded to reveal relevant fields, which can be filled using suggestions or drop-down menus derived from Scheda NU vocabularies. These vocabularies are stored during data entry, streamlining the process for the compiler by guiding label interpretation and word selection.

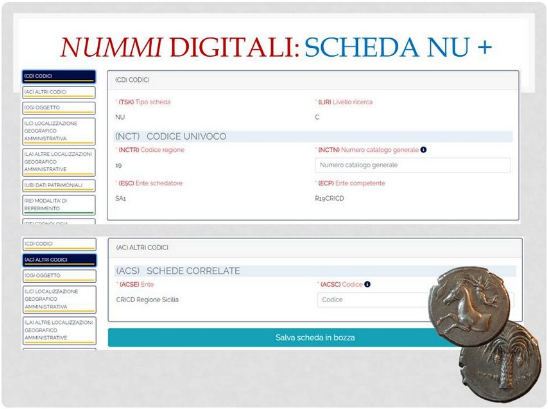

(a)

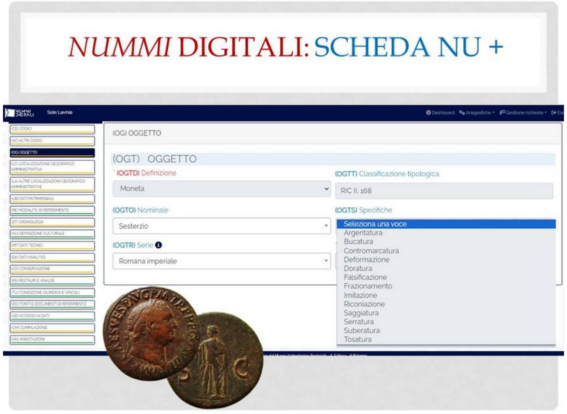

(b)

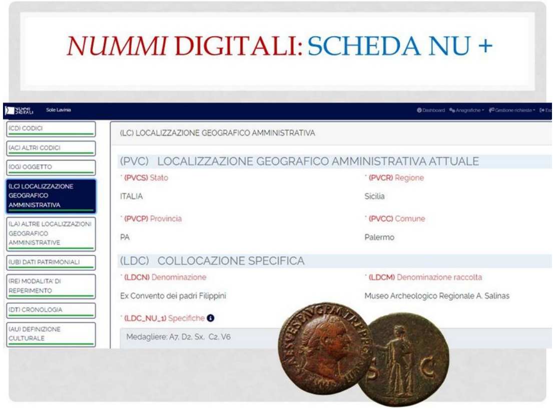

(c)

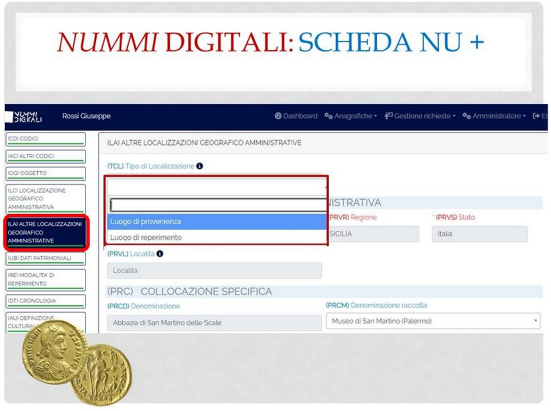

(d)

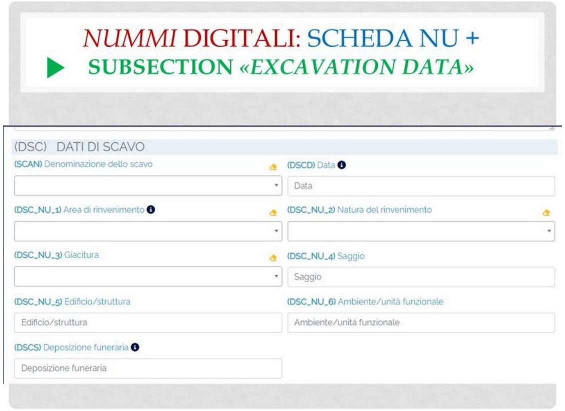

(e)

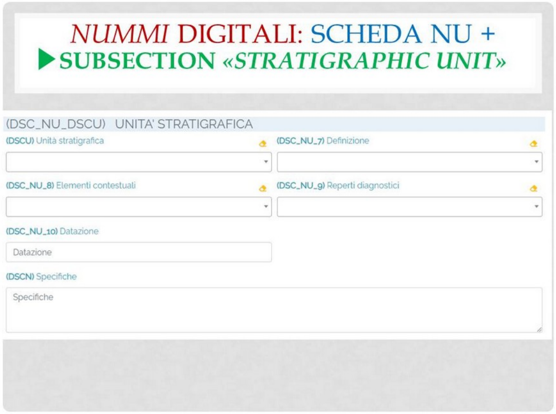

(f)

Figure B: (a) the first two sections: “Codes” (CD-Codici) and “Other codes” (AC-Altri codici); (b) the section “Object” (OGT-Oggetto); (c) the “Administrative geographical location” section (LC-Localizzazione geografico amministrativa); (d) the “Other Administrative geographical locations” section (LA-Altre

localizzazioni geografico amministrative); (e) the “Excavation Data” subsections (DSC-Dati di scavo); (f) the “Stratigraphic Unit” subsections (DSC_NU_DSCU-Unità stratigrafica).
