# Supplementary material for: Nummi Digitali: A pioneering multimodal platform for numismatic heritage
Source: PLoS One. 2025 Oct 3;20(10):e0332151. doi: 10.1371/journal.pone.0332151 (PMC12494253; doi:10.1371/journal.pone.0332151)
Supplement: S4 Appendix — This section describes the methodologies used for digital acquisition, including photogrammetry and structured-light 3D laser scanning, as applied to ancient coins. It discusses instrumentation, setups, resolution strategies, and limitations, with reference to recent literature and comparative approaches. Supplementary S3 Fig documents both scanning systems used: (a) laser scanning with ATOS Q and GOM ROT 350; (b) macro-photogrammetry with Canon EOS 7D and Leica lens. (PDF) [file pone.0332151.s004.pdf]

S4 Appendix. 3D Imaging

The state of the art in digitalization

Thanks to advancements in imaging technologies and computational methods, a wide range of methodologies for the digitization of ancient coins has been refined ([1],[2],[3],[4]). Among the metrology-based methods, photogrammetry and laser scanning have emerged as leading state-of-the-art techniques for generating image-based 3D virtual models.

Photogrammetry is an algorithm-based technique that generates precise and realistic 3D models by analyzing multiple overlapping photographs, calculating depth and geometry, and digitally reconstructing the object's shape and dimensions.

In contrast, 3D Laser Scanning is a non-contact, non-destructive technology that uses laser light to capture an object's geometry. The scanner creates "point clouds," which are data points representing the surface of the object. This process accurately translates the exact size and shape of the physical object into a detailed 3D digital model.

Morris et al. 2022 ([5]) highlights the feasibility of 3D capture for ancient coins, emphasizing the balance between cost and benefit in the process of model generation. MacDonald et al. (2017) ([6]) experimented with a method to improve the spatial resolution of 3D digital models of Roman coins by combining photometric techniques with laser scanning, resulting in a Digital Elevation Map (DEM). Along with photogrammetry, other techniques have been also employed to digitize ancient coins with multi-modal approaches. Hess et al. 2018 ([7]) applied multi-modal imaging techniques to document Roman silver denarii, highlighting the importance of combining different analytical methods to achieve comprehensive visualizations of coin features.
Zambanini et al. 2010 ([8]) demonstrated the successful scanning of historical coins using high-accuracy active stereo scanners, highlighting the utility of 3D models for coin measurement. Additionally, the use of neutron tomography and diffraction studies has provided insights into the material composition and degradation of ancient coins, further enriching our understanding of their historical context ([9]). Despite these valuable efforts, challenges remain in implementing comprehensive systems that ensure accessibility to high-definition images or 360° 3D models, along with contextual information, while engaging a diverse audience—from general users to specialists—through multi-layered content. At the same time, none of the mentioned experiments have been developed to encompass an entire museum collection, to create a fully accessible digital project for numismatic heritage.

3D acquisition.

The photographic set-up is shown in **Figure C-D**.

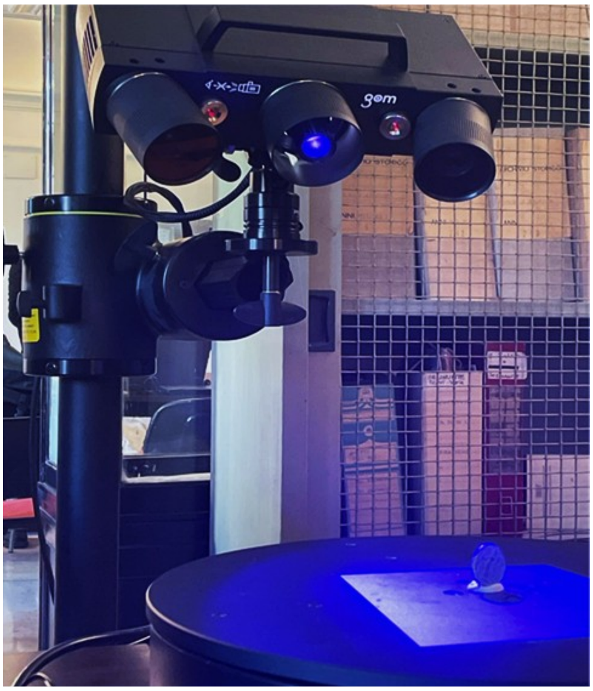

Figure C: Laser scanner Atos Q by GOM (Zeiss), used on site in combination with the GOM ROT 350 rotation table; (b) Macrophotogrammetry: in situ experimental setup including a Canon EOS 7D camera, fixed on a tripod; Macro Lens (Leitz Leica, 1:2.8/60mm) – 1:1 ratio; automatic turntable placed inside a lightbox; printed markers (used as control points).

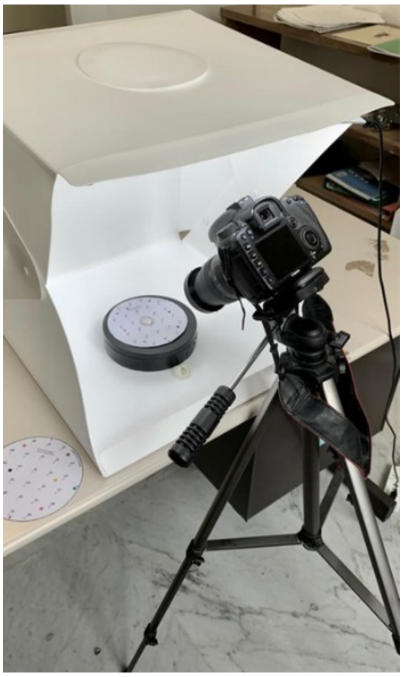

Figure D: Macro-photogrammetry: the experimental setup includes a Canon EOS 7D camera, fixed on a tripod mounting a macro lens (Leitz Leica, 1:2.8/60mm) – 1:1 ratio. The scene includes an automatic turntable placed inside a lightbox and printed markers (used as control points).

References

[1] Ponterio RC, Castrizio D, Renda V, Giuffrida D. Applicazioni di micro-profilometria laser e modellazione 3D per lo studio di due reperti numismatici provenienti da Reggio Calabria. In: Puglisi M, Mondello C, editors. *Acts of "The 8th Joint Meeting of ECFN and nomisma.org on Coin Finds and Digital Numismatics", University of Messina, May 2–4, 2019*. Messina: Messina University Press; 2024. doi: https://doi.org/10.13129/979-12-80899-12-5.

[2] Karami A, Menna F, Remondino F. Combining photogrammetry and photometric stereo to achieve precise and complete 3D reconstruction. Sensors. 2022;22:8172. doi: https://doi.org/10.3390/s22218172.

[3] Palma G, et al. Telling the story of ancient coins by means of interactive RTI images visualization. In: Earl G, Sly T, Wheatley D, Romanowska I, Papadopoulos C, Murrieta-Flores P, Chrysanthi A, editors. *Archaeology in the Digital Era: Papers from the 40th Annual Conference of Computer Applications and Quantitative Methods in Archaeology (CAA)*. Amsterdam: Amsterdam University Press; 2014. pp. 177–185. doi: https://doi.org/10.1017/9789048519590.019.

[4] Jarrett J, Zambanini S, Huber-Mörk R, Felicetti A. Coinage, digitization, and the World-Wide Web: Numismatics and the COINS Project. In: Nelson B, Terras M, editors. *Digitizing Medieval and Early Modern Material Culture*. Toronto: Iter, Inc. and the Arizona Center for Medieval and Renaissance Studies; 2012. pp. 455–485. Available from: http://hdl.handle.net/20.500.12708/27745

[5] Morris G, Emmitt J, Armstrong J. Depth and dimension: Exploring the problems and potential of photogrammetric models for ancient coins. J Comput Appl Archaeol. 2022;5:112–122. doi: https://doi.org/10.5334/jcaa.99.

[6] MacDonald L, Moitinho de Almeida V, Hess M. Three-dimensional reconstruction of Roman coins from photometric image sets. J Electron Imaging. 2017;26:011017-1–20. doi: https://doi.org/10.1117/1.JEI.26.1.011017.

[7] Hess M, MacDonald LW, Valach J. Application of multi-modal 2D and 3D imaging and analytical techniques to document and examine coins on the example of two Roman silver denarii. Herit Sci. 2018;6:5. doi: https://doi.org/10.1186/s40494-018-0169-2.

[8] Zambanini S, Schlapke M, Hödlmoser M, Kampel M. 3D acquisition of historical coins and its application area in numismatics. In: Stork D, Coddington J, Bentkowska-Kafel A, editors. *Computer Vision and Image Analysis of Art*. Bellingham (WA): SPIE & IS&T – The Society for Imaging Science and Technology; 2010. doi: https://doi.org/10.1117/12.840203.

[9] Bakirov B, Saprykina I, Kichanov S, Mimokhod R, Sudarev N, Kozlenko D. Phase composition and its spatial distribution in antique copper coins: Neutron tomography and diffraction studies. J Imaging. 2021;7:129. doi: https://doi.org/10.3390/jimaging7080129.
