# Supplementary material for: Nummi Digitali: A pioneering multimodal platform for numismatic heritage
Source: PLoS One. 2025 Oct 3;20(10):e0332151. doi: 10.1371/journal.pone.0332151 (PMC12494253; doi:10.1371/journal.pone.0332151)
Supplement: S5 Appendix — This file presents the full results of XRF analyses performed on the selected coins, organized in three tables. Table S2 reports Au, Ag, Cu concentrations and carat values for gold and electrum coins; Table S3 shows Ag and Cu content in silver coins; Table S4 details multi-elemental data for copper-based specimens. Anomalies due to subaeration or high Pb content are noted where applicable. (PDF) [file pone.0332151.s005.pdf]

S5 Appendix. XRF data

Table B - Composition of gold and electrum coins.

	% wt.			
**Coin**	**Ag**	**Au**	**Cu**	**Carats**
No. 14537	8 ± 1	92.9 ± 0.9	0.2 ± 0.1	22.3 ± 0.2
No. 51735	0.14 ± 0.01	99.74 ± 0.03	-	23.94 ± 0.01

Table C - Composition of silver coins

	%wt.	
**coin**	**Ag**	**Cu**
No. 26169	95.3±0.8	3.7±0.7
No. 26259	98.6±0.1	1.2±0.1
No. 26117	99.58±0.06	0.21±0.03
No. 26173	99.1±0.2	0.6±0.2
No. 26249	97± 1	1.8±0.2
No. 26103	99.67±0.05	0.15±0.05
No. 9286	98±1	1.0±0.5
No. 26078	99.2±0.3	0.5±0.3
No. 26232	99.41±0.08	0.1±0.1
No. 65413	93.0±0.6	6.2±0.6

For coin No. 26450 the calculation of concentration was not possible because it is a *subaeratus* exemplar.

Table D - Composition of copper-based coins.

	%wt.							
**coin**	**Fe**	**Cu**	**Zn**	**As**	**Pb**	**Bi**	**Sn**	**Sb**
No. 64093	0.07±0.03	67±3	0.05±0.02	1.5±0.1	24±3	0.12±0.01	5.2±0.6	0.13±0.05
No. 9509	0.24±0.06	73±5	0.09±0.01	1.4±0.4	15±7	0.6±0.2	7±1	0.23±0.03

For the coin No. 10010 the calculation of concentration was not possible for the high lead content, over the calibration curve.
