# Supplementary material for: Nummi Digitali: A pioneering multimodal platform for numismatic heritage
Source: PLoS One. 2025 Oct 3;20(10):e0332151. doi: 10.1371/journal.pone.0332151 (PMC12494253; doi:10.1371/journal.pone.0332151)
Supplement: S6 Appendix — This section summarizes the key innovations and results of the Nummi Digitali pilot, including the implementation of a SaaS-based infrastructure, integration of ICCD-compliant metadata with 3D and analytical content, and the digitization of a representative numismatic corpus. (PDF) [file pone.0332151.s006.pdf]

S6 Appendix. Results

The “*Nummi* Digitali” project has achieved the following results and innovations, with a strong impact on the digitization and accessibility of numismatic collections. Among all, we also highlight:

- Efficient back-end system: facilitated the collection, cataloging, and conservation of numismatic information according to ICCD NU Cataloging Standard and the new NU+.
- SaaS configuration: the platform is configured as Software as a Service (SaaS), offering simple, intuitive features accessible entirely from the web.
- User-friendly design: designed to be user-friendly, it does not require specific IT knowledge for management or for using standard XML path export tools.
- Robust infrastructure: currently deployed on a private cloud by the qualified Cloud Service Provider (CSP) ACN (Italian National Cybersecurity Agency) OVH Srl, with plans for certification as a SaaS service on the ACN Marketplace.
- First digitization: the first digitization of the numismatic collection at the "Salinas" Archaeological Museum was achieved.
- Enhanced accessibility**:** high-resolution digital models were created to allow both experts and general users to view and interact with rare coins from different eras within an immersive virtual environment.

The “*Nummi* Digitali” project has successfully demonstrated how a digital multimodal system can enhance the study, improve the management and dissemination of numismatic heritage through the integration of structured data, high-resolution 3D modeling and archaeometric analysis.

The methodological framework developed in “*Nummi* Digitali” has far-reaching implications beyond numismatics, with potential applications in other cultural heritage and museum institutions. The integration of LOD-driven metadata, 3D digitization and analysis sets a new standard for the digitization of ancient artifacts, paving the way for:

- wider adoption of multimodal digitization in the field of cultural heritage;
- cross-disciplinary research initiatives, promoting collaborations between numismatists, material scientists and data scientists;
- enhanced usability to museum collections, democratizing access to scientific datasets and interactive educational tools.
