# Supplementary material for: Nummi Digitali: A pioneering multimodal platform for numismatic heritage
Source: PLoS One. 2025 Oct 3;20(10):e0332151. doi: 10.1371/journal.pone.0332151 (PMC12494253; doi:10.1371/journal.pone.0332151)
Supplement: S7 Appendix — This file details the specific roles of each author in drafting selected sections of the main article and the Supporting Information (PDF) [file pone.0332151.s007.pdf]

S7 Appendix. Author Contributions

1. Main manuscript: L.S. is the author of Introduction; Materials and Methods; Platform architecture; Results: Impact of “Nummi Digitali” project on the numismatic collection of the "A. Salinas" Archeological Museum in Palermo; Discussion. **R.C.P.** and **D.G.** carried out all 3D imaging acquisitions and subsequent data processing, integrated the 3D models into Sketchfab and the project database, and contributed to the writing of the Introduction, Materials and Methods, Results, Discussion, and Conclusion sections pertaining to 3D modeling and platform integration.
M.L.S. and **F.A.** performed XRF acquisition and data analysis, and were responsible for the sections of the manuscript describing XRF methodology and results.
All authors reviewed and approved the final version of the manuscript.

2. Supplementary Information: **LS** is the author of I.Numismatic Databases; II.1 Material and Methods (II.1. Selection of coins from “A. Salinas” regional Archaeological Museum of Palermo). IV. Results; DG and **RCP** authored Supplementary Section II.3, detailing the methodology and results of the 3D imaging procedures. Maria Luisa Saladino and Francesco Armetta prepared the Supplementary XRF Data section, including spectral acquisition and analytical workflows.
