# Supplementary figures and images for: Nummi Digitali: A pioneering multimodal platform for numismatic heritage
Source: PLoS One. 2025 Oct 3;20(10):e0332151. doi: 10.1371/journal.pone.0332151 (PMC12494253; doi:10.1371/journal.pone.0332151)

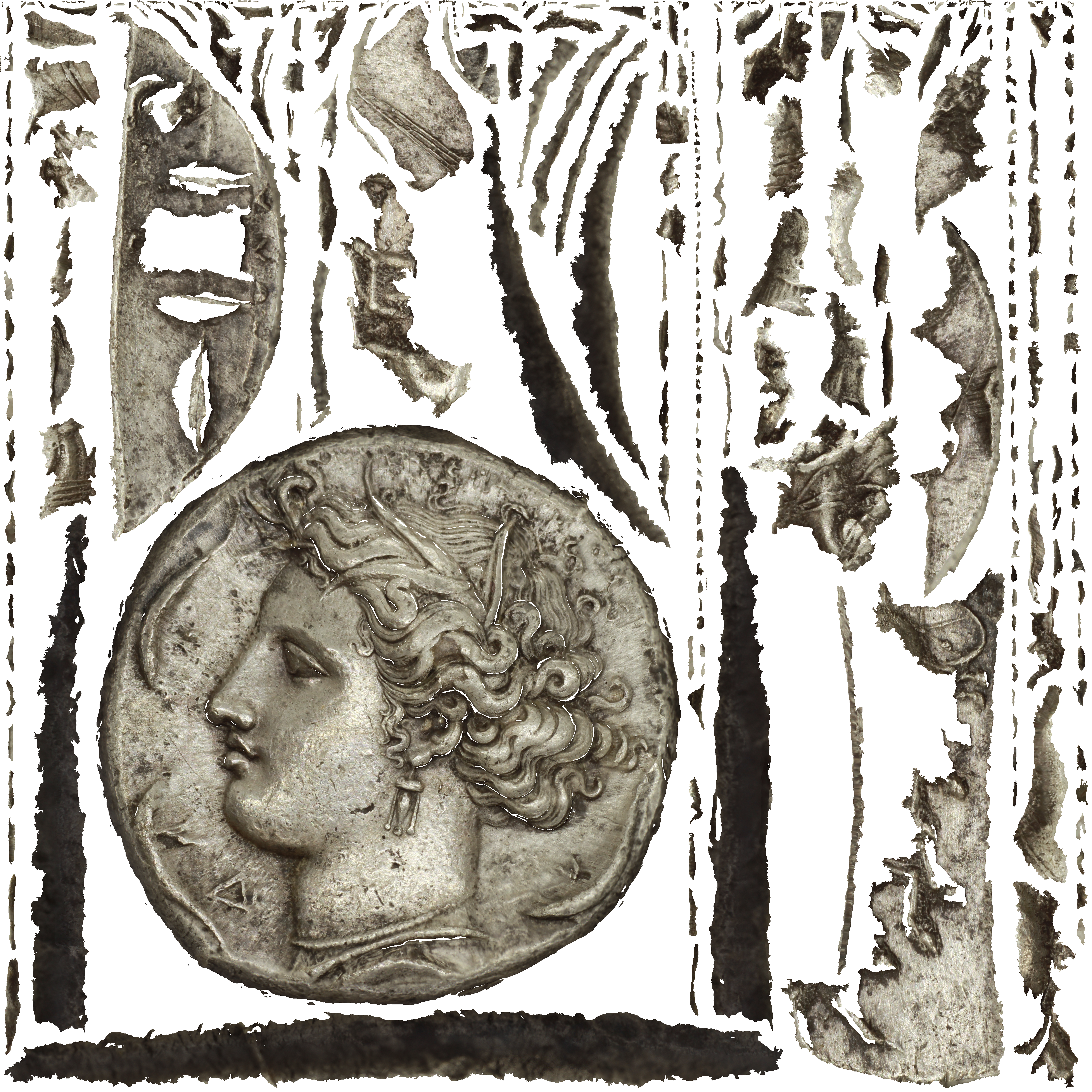

Supplement: S1 File — Integrated 3D model of the coin in 3D format. (ZIP) [file pone.0332151.s008.zip › 26078_2.png]

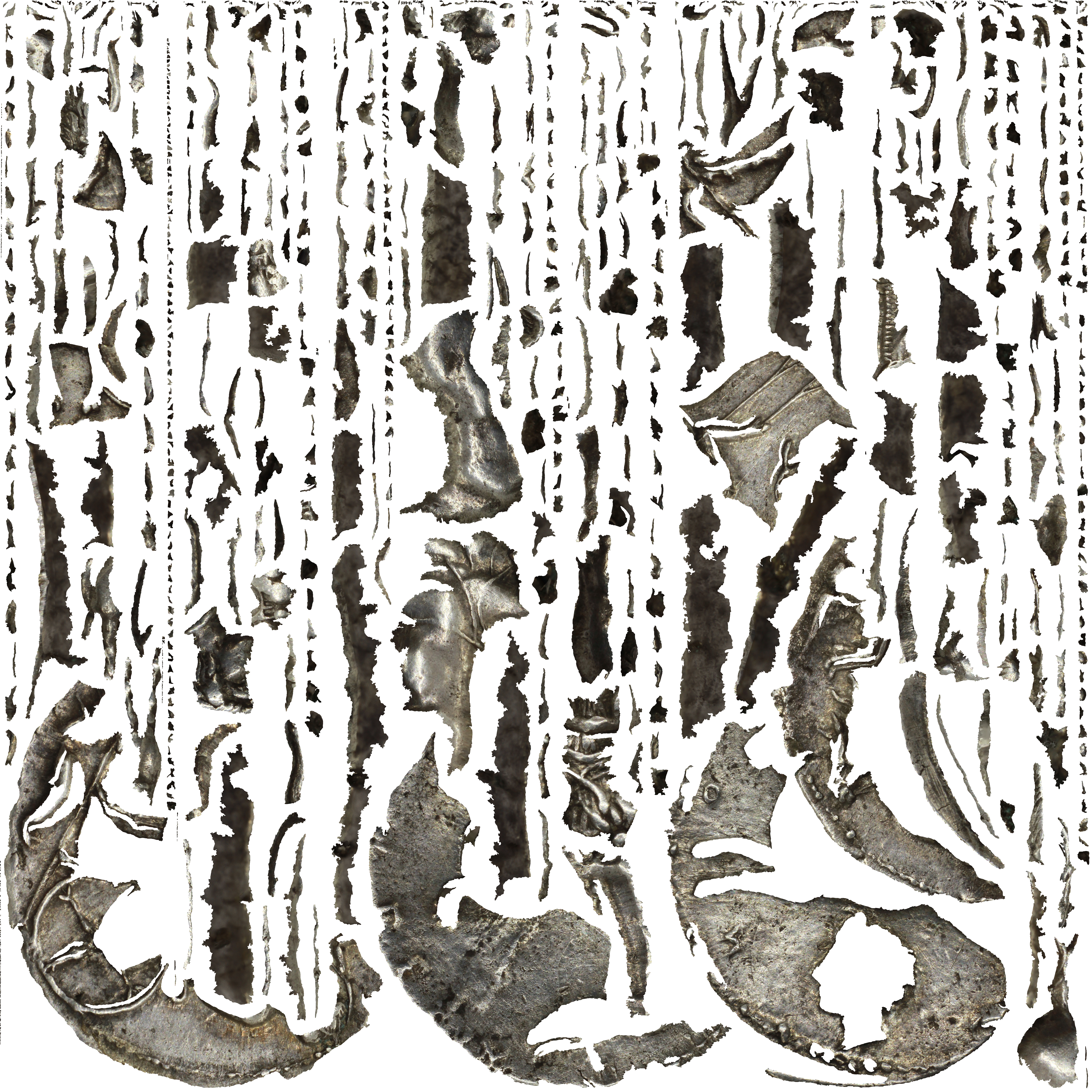

Supplement: S2 File — Integrated 3D model of the coin in 3D format. (ZIP) [file pone.0332151.s009.zip › 26269_2.png]
